# Long-term clinical outcomes of prophylaxis with an rFVIIIFc or rFIXFc in adults aged ≥50 years with hemophilia A or B

**DOI:** 10.1182/bloodadvances.2023012462

**Published:** 2024-07-25

**Authors:** Doris Quon, Shannon Jackson, María Teresa Alvarez-Román, Umer Khan, Sandra Casiano, Margaret V. Ragni, Savita Rangarajan

**Affiliations:** 1 Orthopaedic Hemophilia Treatment Center, Luskin Orthopaedic Institute for Children, Los Angeles, CA; 2 Providence Health Care, St. Paul’s Hospital, Vancouver, BC, Canada; 3 Department of Hematology, University Hospital La Paz, Madrid, Spain; 4 Sanofi, Cambridge, MA; 5 Division of Hematology/Oncology, University of Pittsburgh Medical Center, Pittsburgh, PA; 6 Faculty of Medicine, University of Southampton, Southampton, United Kingdom

**Figure:**
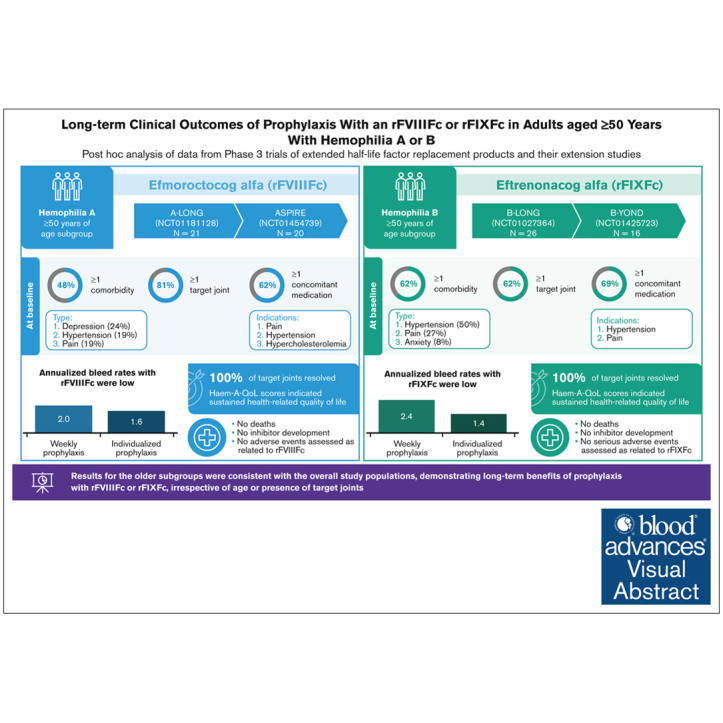

**TO THE EDITOR:**

Older people with hemophilia (PwH) who did not receive prophylaxis from a young age are at an increased risk of developing hemophilic arthropathy.^1^, ^2^, ^3^, ^4^, ^5^ Advances in hemophilia care have led to increased life expectancy and resultant challenges in the management of older PwH.^2^^,^^6^, ^7^, ^8^, ^9^ Limited guidance exists for managing this population.^6^^,^^8^

Efmoroctocog alfa, a recombinant factor VIII (FVIII) Fc fusion protein (referred to herein as rFVIIIFc), and eftrenonacog alfa, a recombinant FIX Fc fusion protein (rFIXFc), are extended half-life factor replacements for hemophilia A or B, respectively, with demonstrated long-term safety and efficacy among individuals of all ages; however, there are limited data in older PwH.^10^, ^11^, ^12^, ^13^, ^14^, ^15^, ^16^, ^17^

This post hoc analysis of A-LONG/ASPIRE and B-LONG/B-YOND studies assessed comorbidities and long-term efficacy and safety in those with severe hemophilia aged ≥50 years. Protocols had local ethics board approvals and were conducted in accordance with the International Conference on Harmonization Guidelines for Good Clinical Practice and the Declaration of Helsinki; participants provided informed consent.^10^, ^11^, ^12^, ^13^ A-LONG enrolled previously treated males aged ≥12 years with severe hemophilia A receiving prophylaxis or on on-demand (OD) therapy with ≥12 bleeding events in the 12 months before the study and no history of inhibitors.^10^ B-LONG enrolled previously treated males aged ≥12 years with severe hemophilia B on a prior prophylaxis regimen or with ≥8 bleeding events in the 12 months before the study and no history of inhibitors.^11^ Eligible participants enrolled in respective extension studies (ASPIRE or B-YOND).^12^^,^^13^ Outcomes included annualized bleed rates (ABRs), target joint resolution, modified Hemophilia Joint Health score (mHJHS; A-LONG/ASPIRE), Haemophilia Quality of Life (QoL) Questionnaire for Adults (Haem-A-QoL), factor consumption, cumulative exposure, and safety. Descriptive statistics are presented for participants with an efficacy period. Detailed methods are in supplemental Material. These trials were registered at www.ClinicalTrials.gov (identifiers: NCT01181128, NCT01027364, NCT01454739, and NCT01425723).

There were 21 participants aged ≥50 years in A-LONG, with 20 participating in ASPIRE. At baseline, median age was 57 years, and 81% had target joints (supplemental Table 1). The FVIII regimen before A-LONG was prophylaxis for 29% (6/21) and OD treatment for 71% (15/21). Participants had a median duration of rFVIIIFc treatment of 4.3 years (interquartile range [IQR], 3.1-5.4) and 318 exposure days (IQR, 257-425). In B-LONG, there were 26 participants aged ≥50 years, with 16 participating in B-YOND. The median age at baseline was 56 years, and 62% had target joints (supplemental Table 2). Before B-LONG enrollment, 11 of 26 patients (42%) were treated prophylactically, and 15 of 26 (58%) were treated OD. The median (IQR) duration of rFIXFc treatment was 3.4 years (IQR, 1.0-5.4); 54% had ≥3 years of treatment. The median number of rFIXFc exposure days was 101 (IQR, 44-220).

Approximately half the participants in A-LONG/ASPIRE (10/21 [48%]) and B-LONG/B-YOND (16/26 [62%]) had ≥1 concomitant health condition at baseline (supplemental Tables 1 and 2). Common conditions in participants in A-LONG/ASPIRE were depression (5/21 [24%]), hypertension (4/21 [19%]), and pain (4/21 [19%]), and in B-LONG/B-YOND, they were hypertension (13/26 [50%]) and pain (7/26 [27%]). Comorbidities were similar to those reported previously, highlighting physical and mental health conditions affecting aging PwH.^6^^,^^9^ Prevalence of hypertension reported here is lower than reported elsewhere^6^^,^^18^^,^^19^; in the Hematology Utilization Group Study Part VII (HUGS VII) study, 57% of PwH A/B of all severities aged ≥50 years in the United States had hypertension.^6^

Most participants were taking ≥1 concomitant medication at baseline (A-LONG/ASPIRE, 13/21 [62%]; B-LONG/B-YOND, 18/26 [69%]; supplemental Tables 1 and 2). The most common indications were pain (largely related to arthropathy/joint pain) and hypertension. Acute and chronic pain are common features of hemophilia due to bleeds and/or arthropathy. Older individuals face the burden of both hemophilia- and age-related joint degeneration, likely due to receiving suboptimal hemophilia management in earlier years. Only timely administration of FVIII/FIX early in life has demonstrated efficacy in preserving joint health among individuals with severe hemophilia.^5^^,^^20^ Furthermore, although several participants in this analysis experienced depression or anxiety, no medications were reported, suggesting inadequate treatment for these conditions. Depression is common in older people with physical health conditions; it significantly affects QoL and is associated with poorer treatment outcomes.^21^ It is important that PwH receive effective treatment and support for mental health conditions to cope with the burden of hemophilia- and age-related comorbidities.

Prophylaxis with rFVIIIFc and rFIXFc resulted in low overall and joint ABRs in participants aged ≥50 years, including those with target joints at baseline (Figure 1). As expected, prestudy ABRs were higher in participants treated OD than those with prior prophylaxis. These results are consistent with adults/adolescents in the overall study populations.^10^, ^11^, ^12^, ^13^

**Figure 1.**
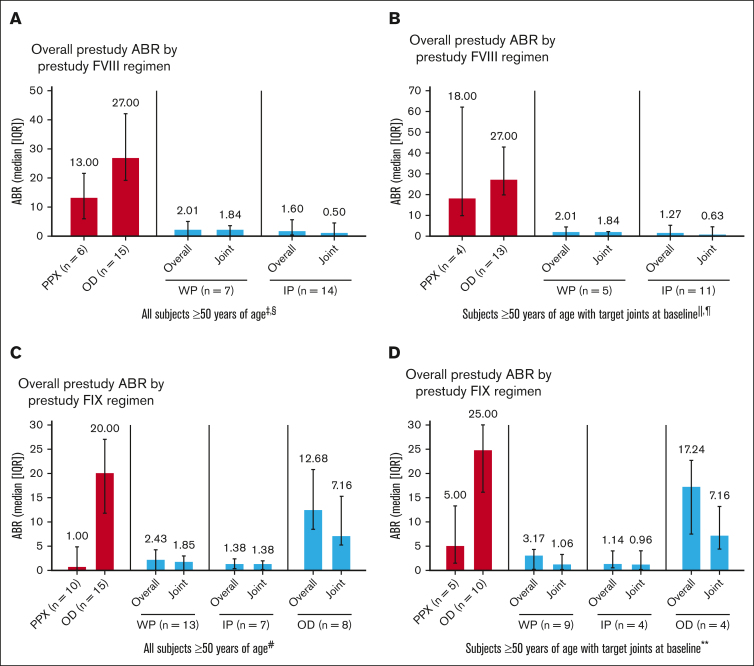
**ABRs by treatment regimen pooled from A-LONG/ASPIRE and B-LONG/B-YOND.** (A) All participants aged ≥50 years in A-LONG/ASPIRE. (B) Participants with target joints at baseline aged ≥50 years in A-LONG/ASPIRE. (C) All participants aged ≥50 years in B-LONG/B-YOND. (D) Participants with target joints at baseline aged ≥50 years in B-LONG/B-YOND. ∗Participants could change treatment regimens at any time during ASPIRE/B-YOND and may appear in ≥1 group; they are considered in each treatment regimen they participated in for the duration of time on that regimen; n is the number of participants in the specific treatment regimen and with an efficacy period. ^†^One participant was missing prestudy ABR for the B-LONG study. ^‡^Among 3 participants on modified prophylaxis during the study, the median overall ABR was 4.61 (IQR, 0.64-5.12); median joint ABR was 2.84 (IQR, 0.64-3.07; data not shown in graph). ^§^Among 3 participants receiving OD treatment during the study (data not shown in graph), median overall ABR was 17.40 (IQR, 9.81-81.54); median joint ABR was 13.42 (IQR, 5.89-72.86). ^||^Among 3 participants with target joints at baseline on modified prophylaxis during the study (data not shown in graph), median overall ABR was 4.61 (IQR, 0.64-5.12); median target joint ABR was 2.84 (IQR, 0.64-3.07). ^¶^Among 2 participants with target joints at baseline receiving OD treatment during the study (data not shown in graph), median overall ABR was 49.47 (IQR, 17.40-81.54); median target joint ABR was 43.14 (IQR, 13.42-72.86). ^#^Among 4 participants on modified prophylaxis during the study (data not shown in graph), median overall ABR was 7.07 (IQR, 5.36-8.41); median joint ABR was 3.99 (IQR, 1.24-6.94). ∗∗Among 3 participants with target joints at baseline on modified prophylaxis during the study (data not shown in graph), median overall ABR was 6.44 (IQR, 4.28-7.70); median target joint ABR was 1.56 (IQR, 0.92-6.41). IP, individualized prophylaxis; PPX, prophylaxis; WP, weekly prophylaxis.

Joint health improved in PwH aged ≥50 years. For those always on prophylaxis with an mHJHS at each time point (n = 10), the mean mHJHS was 50.10 (standard deviation [SD], 15.79) at A-LONG baseline and 41.70 (SD, 11.48) at the last available visit in ASPIRE (mean change from baseline, –8.40 [SD, 10.87]). All 42 evaluable target joints in 12 participants from A-LONG/ASPIRE and all 19 in 6 participants from B-LONG/B-YOND resolved with prophylaxis.

For participants always on prophylaxis, the mean changes in total Haem-A-QoL scores from A-LONG/B-LONG baseline to the last visit were –2.1 (SD, 12.11) and –4.9 (SD, 6.83), respectively (Figure 2). The subdomains with the greatest improvement were sports and leisure in A-LONG/ASPIRE and view of yourself in B-LONG/B-YOND. Although there is a difference in the scale and direction of change in Haem-A-QoL domain scores between participants with hemophilia A and B, we hesitate to draw conclusions about these differences related to limited sample size. QoL among PwH worsens with age, highlighting the burden of age-related health conditions.^6^ We report sustained QoL in participants aged ≥50 years on rFVIIIFc/rFIXFc prophylaxis, with improvements in some domains, including physical health and sports and leisure, demonstrating that bleed protection and joint health benefits of prophylaxis may enable older PwH to be more physically active.

**Figure 2.**
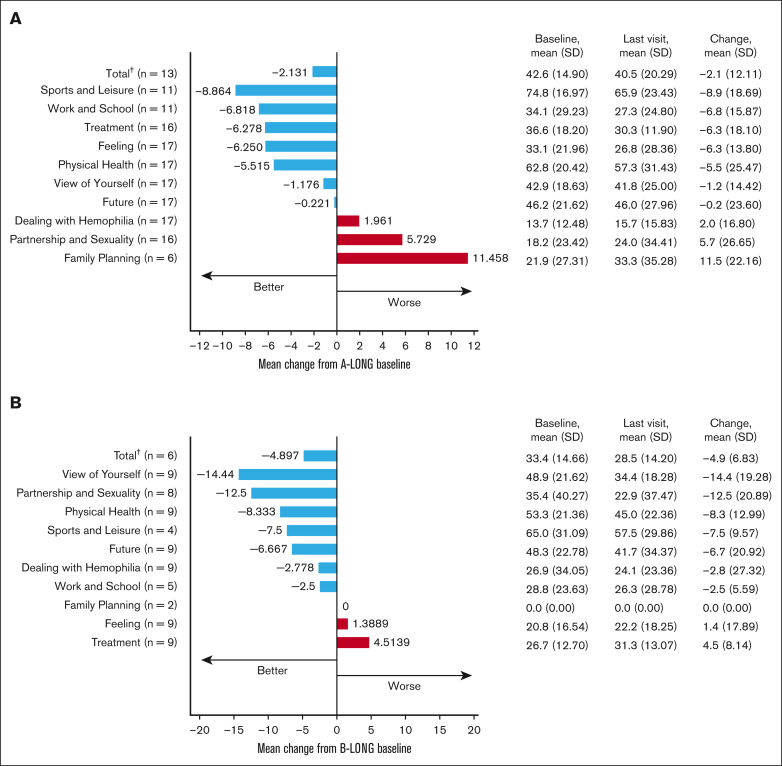
**Health-related QoL in participants aged ≥50 years on rFVIIIFc or rFIXFc prophylaxis.** (A) Change in Haem-A-QoL score∗ from A-LONG baseline to the last visit with a score for participants who were always on a prophylaxis regimen. (B) Change in Haem-A-QoL score∗ from B-LONG baseline to the last study visit with a score for participants who were always on a prophylaxis regimen. ∗Haem-A-QoL scores range from 0 to 100, with higher scores representing a higher impairment in quality of life for each subscore and total score. ^†^Last visit of few categories is not the same as last visit of total category. Hence, the total value is not the sum of respective categories.

Participants on prophylaxis did not require a significant increase in factor dose to maintain the low ABRs observed, with a mean change in dose at the end of A-LONG/B-LONG compared with the end of ASPIRE/B-YOND of 6.5 IU/kg (SD, 10.6) and 3.9 IU/kg (SD, 15.7), respectively (supplemental Table 3).

Safety outcomes were consistent with those of the overall populations of the respective studies.^10^, ^11^, ^12^, ^13^ No new safety concerns, deaths, or inhibitor development occurred in the older subpopulation. Treatment-emergent adverse events (TEAEs) were reported in 20 of 21 (95%) and 23 of 26 participants (89%) aged ≥50 years in A-LONG/ASPIRE and B-LONG/B-YOND, respectively (supplemental Table 4). Most TEAEs were mild or moderate and as expected for this population (supplemental Tables 4-6). Serious TEAEs were reported in 8 participants in A-LONG/ASPIRE and 14 participants in B-LONG/B-YOND. No serious adverse events were related to either rFVIIIFc or rFIXFc treatment (supplemental Tables 4, 7, and 8).

Limitations of this analysis include the relatively small sample size, exclusion of patients with certain comorbidities/medications that may be more common in older PwH, limited diversity potentially affecting the spectrum of comorbidities, and that concomitant conditions/medications were only recorded at baseline, barring long-term assessments. This analysis also did not assess the impact of hepatitis C virus or HIV infection.

Results for the subgroups of those aged ≥50 years are consistent with the overall study populations, suggesting that rFVIIIFc and rFIXFc provide long-term clinical benefits irrespective of age or presence of target joints in PwH. These results highlight that the prevalence of concomitant conditions and reliance on medications in older PwH predominantly stems from the burden of pain and chronic arthropathy, underscoring the necessity of initiating prophylaxis at an early age. Further research is essential to developing best practices for the management of aging PwH.

**Conflict-of-interest disclosure:** D.Q. reports consultancy and/or speaker’s bureau work for Bayer, Sanofi, Genentech, Novo Nordisk, Octapharma, BioMarin Pharmaceutical, CSL Behring, and Takeda. S.J. reports consultancy for Bayer, Roche, Sanofi, and Takeda. M.T.A.-R. has received honoraria for consulting from Takeda, Bayer, BioMarin Pharmaceutical, CSL Behring, Grifols, Novo Nordisk, Sobi, Roche, Octapharma, and Pfizer; and funds for research from Takeda, Bayer, Grifols, Novo Nordisk, and Roche. S.C. and U.K. are employees of Sanofi and may hold shares and/or stock options in the company. M.V.R. reports personal fees/other from Alnylam, BeBio, BioMarin Pharmaceutical, *Blood Advances* Editorial Board, Foundation for Women & Girls with Blood Disorders, Hemab Therapeutics (HEMAB), Hemostasis & Thrombosis Research Society (HTRS), Institute for Clinical and Economic Review (ICER), Sanofi, Spark, Takeda, and University of Cincinnati, along with nonfinancial support from Takeda, outside the submitted work. S.R. is a consultant for Reliance Life Sciences.

## Supplementary Material

Supplemental Methods, References, and Tables

## References

[1] Srivastava A, Santagostino E, Dougall A (2020). WFH guidelines for the management of hemophilia. Haemophilia.

[2] Canaro M, Goranova-Marinova V, Berntorp E (2015). The ageing patient with haemophilia. Eur J Haematol.

[3] Oldenburg J, Kulkarni R, Srivastava A (2018). Improved joint health in subjects with severe haemophilia A treated prophylactically with recombinant factor VIII Fc fusion protein. Haemophilia.

[4] Manco-Johnson MJ, Abshire TC, Shapiro AD (2007). Prophylaxis versus episodic treatment to prevent joint disease in boys with severe hemophilia. N Engl J Med.

[5] Warren BB, Thornhill D, Stein J (2020). Young adult outcomes of childhood prophylaxis for severe hemophilia A: results of the joint outcome continuation study. Blood Adv.

[6] Curtis R, Manco-Johnson M, Konkle BA (2022). Comorbidities, health-related quality of life, health-care utilization in older persons with Hemophilia-Hematology Utilization Group Study part VII (HUGS VII). J Blood Med.

[7] Angelini D, Sood SL (2015). Managing older patients with hemophilia. Hematology.

[8] Reding MT, Pabinger I, Holme PA (2023). Efficacy and safety of damoctocog alfa pegol prophylaxis in patients ⩾40 years with severe haemophilia A and comorbidities: post hoc analysis from the PROTECT VIII study. Ther Adv Hematol.

[9] Steen Carlsson K, Winding B, Astermark J (2021). People with haemophilia including female carriers in Nordic countries die at an earlier age and have significant co-morbidities. Haemophilia.

[10] Mahlangu J, Powell JS, Ragni MV (2014). Phase 3 study of recombinant factor VIII Fc fusion protein in severe hemophilia A. Blood.

[11] Powell JS, Pasi KJ, Ragni MV (2013). Phase 3 study of recombinant factor IX Fc fusion protein in hemophilia B. N Engl J Med.

[12] Nolan B, Mahlangu J, Pabinger I (2020). Recombinant factor VIII Fc fusion protein for the treatment of severe haemophilia A: final results from the ASPIRE extension study. Haemophilia.

[13] Pasi KJ, Fischer K, Ragni M (2020). Long-term safety and sustained efficacy for up to 5 years of treatment with recombinant factor IX Fc fusion protein in subjects with haemophilia B: results from the B-YOND extension study. Haemophilia.

[14] Fischer K, Kulkarni R, Nolan B (2017). Recombinant factor IX Fc fusion protein in children with haemophilia B (Kids B-LONG): results from a multicentre, non-randomised phase 3 study. Lancet Haematol.

[15] Königs C, Ozelo MC, Dunn A (2022). First study of extended half-life rFVIIIFc in previously untreated patients with hemophilia A: PUPs A-LONG final results. Blood.

[16] Nolan B, Klukowska A, Shapiro A (2021). Final results of the PUPs B-LONG study: evaluating safety and efficacy of rFIXFc in previously untreated patients with hemophilia B. Blood Adv.

[17] Young G, Mahlangu J, Kulkarni R (2015). Recombinant factor VIII Fc fusion protein for the prevention and treatment of bleeding in children with severe hemophilia A. J Thromb Haemost.

[18] Badescu MC, Badulescu OV, Butnariu LI (2022). Cardiovascular risk factors in patients with congenital hemophilia: a focus on hypertension. Diagnostics (Basel).

[19] von Drygalski A, Kolaitis NA, Bettencourt R (2013). Prevalence and risk factors for hypertension in hemophilia. Hypertension.

[20] Oldenburg J, Zimmermann R, Katsarou O (2015). Controlled, cross-sectional MRI evaluation of joint status in severe haemophilia A patients treated with prophylaxis vs. on demand. Haemophilia.

[21] Sivertsen H, Bjørkløf GH, Engedal K, Selbæk G, Helvik AS (2015). Depression and quality of life in older persons: a review. Dement Geriatr Cogn Disord.

