# Supplementary material for: Long-term clinical outcomes of prophylaxis with an rFVIIIFc or rFIXFc in adults aged ≥50 years with hemophilia A or B
Source: Blood Adv. 2024 Jul 25;8(18):4751–5. doi: 10.1182/bloodadvances.2023012462 (PMC11414653; doi:10.1182/bloodadvances.2023012462)
Supplement: Supplemental Methods, References, and Tables [file BLOODA_ADV-2023-012462-mmc1.pdf]

Supplementary Material

Contents

Methods 2

A-LONG/ASPIRE study design: 2

B-LONG/B-YOND study design: 2

Exclusion criteria: 2

Target joint definition: 3

Efficacy period definition: 3

References: 3

Table S1. Baseline characteristics and comorbidities and medications of participants ≥50 years of age in A-LONG and ASPIRE studies* 4

Table S2. Baseline characteristics and comorbidities and medications of participants ≥50 years of age in B-LONG and B-YOND studies* 6

Table S3. Factor consumption in participants ≥50 years of age only ever on prophylaxis in A-LONG/ASPIRE and B-LONG/B-YOND 8

Table S4. Safety in participants ≥50 years of age in A-LONG/ASPIRE and B-LONG/B-YOND 9

Table S5. Treatment-emergent adverse events in participants ≥50 years of age during A-LONG/ASPIRE 10

Table S6. Treatment-emergent adverse events in participants ≥50 years of age during B-LONG/B-YOND 14

Table S7. Serious treatment-emergent adverse events in participants ≥50 years of age during A-LONG/ASPIRE 18

Table S8. Serious treatment-emergent adverse events in participants ≥50 years of age during B-LONG/B-YOND 19

Methods

A-LONG/ASPIRE study design:

A-LONG enrolled previously treated (≥150 exposure days [EDs] to replacement FVIII) males aged ≥12 years with severe hemophilia A (<1 IU/dL endogenous FVIII activity) receiving prophylaxis, or on-demand therapy with a history of ≥12 bleeding events in the 12 months prior to the study, and no history of inhibitors.[1] Treatment groups included individualized prophylaxis (IP; rFVIIIFc 25 IU/kg on Day 1 and 50 IU/kg on Day 4, followed by 25-65 IU/kg every 3-5 days), weekly prophylaxis (WP; rFVIIIFc 65 IU/kg every 7 days), and OD treatment (bleed-dependent dosing), with intervals and dosing adjusted in the IP and WP groups to target 1%-3% trough levels.[1] Eligible participants from A-LONG could enroll in ASPIRE.[2] Treatment groups in ASPIRE included IP (rFVIIIFc 25-65 IU/kg every 3-5 days or twice weekly), WP (rFVIIIFc 65 IU/kg every 7 days), modified prophylaxis (MP; personalized dosing for patients who required more individualized regimens), and OD treatment.[2] Participants could switch regimens at any time and may appear in ≥1 treatment group in ASPIRE, at the investigator's discretion.

B-LONG/B-YOND study design:

B-LONG enrolled previously treated (≥100 EDs to replacement FIX) males aged ≥12 years with severe hemophilia B (≤2 IU/dL endogenous FIX activity), on a prior prophylaxis regimen or with a history of ≥8 bleeding events in the 12 months prior to the study, and no history of inhibitors.[3] Treatment groups in B-LONG included IP (rFIXFc 100 IU/kg every 10 days to start), WP (rFIXFc 50 IU/kg to start every 7 days), and OD (rFIXFc 20-100 IU/kg as required).[3] Intervals and doses were adjusted for IP and WP groups as needed to maintain a trough level of 1%-3% above baseline.[3] Eligible participants from B-LONG could enroll in B-YOND.[4] Treatment groups in B-YOND were IP (rFIXFc 100 IU/kg every 8-16 days or twice monthly), WP (rFIXFc 20-100 IU/kg every 7 days), MP (personalized dosing for patients who required more individualized regimens), and OD.[4] Patients could switch regimens during B-YOND at the investigator's discretion.

Exclusion criteria:

Exclusion criteria in the studies included abnormal renal function; active hepatic disease; any concurrent clinically significant major disease that, in the opinion of the Investigator, makes the participant unsuitable for enrollment; uncontrolled HIV infection and concurrent systemic

treatment with immunosuppressant drugs (exceptions: ribavirin, treatment of HCV and HIV and/or systemic steroids and/or inhaled steroids).[1,3]

Target joint definition:

A target joint was defined as a major joint (eg, knee, ankle, elbow, hip, shoulder, and wrist) in which repeated spontaneous bleeds occurred (≥3 bleeding episodes into the same joint in a consecutive 6-month period [A-LONG/ASPIRE] or 3-month period [B-LONG/B-YOND]). Target joints were considered evaluable if there had been ≥12 months consecutive follow-up and no surgery on the target joint within the 12 months since the start of follow-up. Target joint resolution was defined as <3 spontaneous bleeds occurring in the joint over a 12-month period.

Efficacy period definition:

Efficacy periods for each treatment regimen were the sum of time intervals during which patients received rFVIIIFc/rFIXFc according to the specific regimen, excluding major and minor surgical/rehabilitation periods and large injection intervals (>28 days and >42 days between any 2 adjacent injections in a prophylactic regimen for rFVIIIFc and rFIXFc, respectively).

References:

1. Mahlangu J, Powell JS, Ragni MV, et al. Phase 3 study of recombinant factor VIII Fc fusion protein in severe hemophilia A. *Blood*. 2014;123(3):317-325.
2. Nolan B, Mahlangu J, Pabinger I, et al. Recombinant factor VIII Fc fusion protein for the treatment of severe haemophilia A: Final results from the ASPIRE extension study. *Haemophilia*. 2020;26(3):494-502.
3. Powell JS, Pasi KJ, Ragni MV, et al. Phase 3 study of recombinant factor IX Fc fusion protein in hemophilia B. *N Engl J Med*. 2013;369(24):2313-2323.
4. Pasi KJ, Fischer K, Ragni M, et al. Long-term safety and sustained efficacy for up to 5 years of treatment with recombinant factor IX Fc fusion protein in subjects with haemophilia B: Results from the B-YOND extension study. *Haemophilia*. 2020;26(6):e262-e271.

Table S1. Baseline characteristics and comorbidities and medications of participants ≥50 years of age in A-LONG and ASPIRE studies*

Characteristic	A-LONG (N=21)	ASPIRE[†] (N=20)
**Age, years**		
Median (range)	57 (50-65)	57 (50-66)
Mean (SD)	57.24 (4.24)	57.95 (4.44)
**Race, n (%)**		
White	16 (76.2)	16 (80.0)
Asian	5 (23.8)	4 (20.0)
**FVIII genotype, n (%)**		
Nonsense	8 (38.1)	8 (40.0)
Intron 22 inversion	5 (23.8)	5 (25.0)
Frameshift	4 (19.1)	4 (20.0)
Intron 1 inversion	2 (9.5)	2 (10.0)
Missense	1 (4.8)	1 (5.0)
Unknown	1 (4.8)	0 (0.0)
**Weight, kg**		
Median (range)	70.0 (50.0–100.0)	71.2 (51.0–103.4)
Mean (SD)	71.5 (13.7)	71.2 (14.3)
**BMI, kg/m^2**		
Median (range)	24.7 (19.0–31.9)	25.1 (17.6–32.5)
Mean (SD)	24.6 (4.4)	24.6 (4.6)
**BMI (as per WHO standards), n (%)**		
Underweight (BMI <18.5)	0	2 (10.0)
Healthy weight (BMI 18.5–<25)	11 (52.4)	7 (35.0)
Overweight (BMI 25–<30)	6 (28.6)	9 (45.0)
Obesity (BMI ≥30)	4 (19.1)	2 (10.0)
**Prestudy FVIII regimen, n (%)[‡]**		
Prophylaxis	6 (28.6)	17 (85.0)
Weekly prophylaxis	N/A	4 (20.0)
Individualized prophylaxis	N/A	13 (65.0)
On-demand	15 (71.4)	3 (15.0)
**Baseline target joint involvement, n (%)**		
Yes	17 (81.0)	16 (80.0)
No	4 (19.1)	4 (20.0)
**Comorbidities and medications**	**A-LONG/ASPIRE (N=21)**	
**Number of comorbidities, n (%)**		
0	11 (52.4)	
1	5 (23.8)	
2	2 (9.5)	
3	3 (14.3)	
≥1	10 (47.6)	
≥2	5 (23.8)	
**Comorbidities, n (%)**		
Depression	5 (23.8)[§]	
Hypertension	4 (19.1)	

Pain	4 (19.1)[ǁ]
Anxiety	2 (9.5)
Benign colon tumor	1 (4.8)
Diabetes mellitus	1 (4.8)
Urachal tumor (removed 1985)	1 (4.8)
**Number of concomitant medications, n (%)**	
0	8 (38.1)
1	9 (42.9)
2	3 (14.3)
3	1 (4.8)
≥1	13 (61.9)
≥2	4 (19.1)
**Concomitant medication indications, n (%)**	
Hypertension	4 (19.1)
Pain	
Joint pain	4 (19.1)[¶]
Arthropathy pain	3 (14.3)[#]
Pain control/relief	3 (14.3)
Chronic pain	1 (4.8)
Arthritis pain	1 (4.8)
Low back pain	1 (4.8)
Hypercholesterolemia	1 (4.8)

BMI, body mass index; FVIII, factor VIII; N/A, not applicable; rFVIIIFc, recombinant factor VIII Fc fusion protein; SD, standard deviation; WHO, World Health Organization.

*Participants who were ≥50 years of age with an efficacy period. Efficacy periods for each treatment regimen were the sum of time intervals during which participants received rFVIIIFc according to the specific regimen, excluding major and minor surgical/rehabilitation periods and large injection intervals (>28 days between any 2 adjacent injections in a prophylactic rFVIIIFc regimen). [†]In ASPIRE, participants could change treatment regimen at any time and may appear in ≥1 treatment regimen. [‡]Prestudy regimen for ASPIRE refers to the FVIII regimen in A-LONG. [§]Includes 'Depression' (n=4) and 'Depression, on medication' (n=1). [ǁ]Includes 'chronic pain' (n=1); 'chronic pain due to arthropathy' (n=1); 'chronic pain syndrome, on medication' (n=1); and 'low back pain' (n=1). [¶]Includes 'joint pain management'; 'joint pain secondary to hemophilia A'; 'severe chronic pain in joints'; and 'ankle pain'. [#]Includes 'arthropathy pain'; 'chronic pain due to arthropathy'; and 'hemophilic arthropathy pain'.

Table S2. Baseline characteristics and comorbidities and medications of participants ≥50 years of age in B-LONG and B-YOND studies*

Characteristic	B-LONG (N=26)	B-YOND[†] (N=16)
**Age, years**		
Median (range)	56 (50-71)	57.5 (52-63)
Mean (SD)	56.65 (5.85)	57.44 (3.81)
**Race, n (%)**		
White	17 (65.4)	9 (56.3)
Asian	6 (23.1)	6 (37.5)
Black	2 (7.7)	1 (6.3)
American Indian or Alaska Native	1 (3.9)	0 (0.0)
**Weight, kg**		
Median (range)	74.0 (50.0–123.4)	73.7 (50.0–122.5)
Mean (SD)	72.5 (15.6)	76.0 (19.8)
**BMI, kg/m^2**		
Median (range)	25.8 (18.6–36.5)	26.1 (18.6–36.2)
Mean (SD)	25.8 (4.0)	26.2 (5.1)
**BMI (as per WHO standards), n (%)**		
Underweight (BMI <18.5)	0	0
Healthy weight (BMI 18.5–<25)	10 (38.5)	6 (37.5)
Overweight (BMI 25–<30)	13 (50.0)	6 (37.5)
Obesity (BMI ≥30)	3 (11.5)	4 (25.0)
**FIX genotype, n (%)**		
Missense mutation	16 (61.5)	11 (68.8)
Nonsense mutation	6 (23.1)	3 (18.8)
Frameshift	1 (3.9)	0 (0.0)
Large deletions	3 (11.5)	2 (12.5)
**Prestudy FIX regimen, n (%)[‡]**		
Prophylaxis	11 (42.3)	12 (75.0)
Weekly prophylaxis	N/A	5 (31.3)
Individualized prophylaxis	N/A	7 (43.8)
On-demand	15 (57.7)	3 (18.8)
Surgery	0 (0.0)	1 (6.3)
**Baseline target joint involvement, n (%)**		
Yes	16 (61.5)	9 (56.3)
No	10 (38.5)	7 (43.8)
**Comorbidities and medications**	**B-LONG/B-YOND (N=26)**	
**Number of comorbidities, n (%)**		
0	10 (38.5)	
1	4 (15.4)	
2	10 (38.5)	
3	0 (0.0)	
4	2 (7.7)	
≥1	16 (61.5)	
≥2	12 (46.2)	
**Comorbidities, n (%)**		
Hypertension	13 (50.0)	
Pain	7 (26.9)[§]	

Anxiety	2 (7.7)[II]
Depression	1 (3.9)
Portal hypertension	1 (3.9)
Pseudotumor, right thigh	1 (3.9)
Toxicity from pegylated interferon and ribavirin therapy for hepatitis C, including – depression	1 (3.9)
Type 2 diabetes	1 (3.9)
Diabetes mellitus	1 (3.9)
Non-insulin dependent diabetes	1 (3.9)
**Number of concomitant medications, n (%)**	
0	8 (30.8)
1	12 (46.2)
2	4 (15.4)
3	2 (7.7)
≥1	18 (69.2)
≥2	6 (23.1)
**Concomitant medication indications, n (%)**	
Hypertension	11 (42.3)
Pain	
Joint pain	7 (26.9)[¶]
Pain due to hemophilic arthropathy	4 (15.4)[#]
Hemarthropic pain associated with breakthrough bleed	1 (3.9)
Chronic pain	1 (3.9)
Hemophilia-related joint pain	1 (3.9)
Hemophilia-related pain	1 (3.9)

BMI, body mass index; FIX, factor IX; N/A, not applicable; rFIXFc, recombinant factor IX Fc fusion protein; SD, standard deviation; WHO, World Health Organization.

*Participants who were ≥50 years of age with an efficacy period. Efficacy periods for each treatment regimen were the sum of time intervals during which participants received rFIXFc according to the specific regimen, excluding major and minor surgical/rehabilitation periods and large injection intervals (>42 days between any 2 adjacent injections in a prophylactic rFIXFc regimen). [†]In B-YOND, participants could change treatment regimen at any time and may appear in ≥1 treatment regimen. [‡]Prestudy regimen for B-YOND refers to the FIX regimen in B-LONG. [§]Includes ‘back pain’ (n=1); ‘bilateral hip decreased range of motion and pain’ (n=1); ‘chronic pain’ (n=1); ‘hemophilia-related pain’ (n=1); ‘low back pain’ (n=1); ‘pain, restriction of motion’ (n=2); ‘pain, swelling, restriction of motion’ (n=1); ‘pain, swelling, restriction of motion, increased temperature’ (n=1); ‘abdominal pain, possible renal colic’ (n=1). [II]Includes participants with ‘anxiety’ (n=1) and ‘stress and anxiety’ (n=1). [¶]Includes ‘arthritic pain’; ‘arthritic, toothache, and joint pain’; ‘general joint pain (primarily left knee)’; ‘in case of joint pain’; ‘joint pain’; ‘pain for hip pain’, ‘joint pain, osteoarthritis’. [#]Includes ‘pain for hemophilic arthropathy’; ‘pain secondary to arthropathy’; ‘hemarthropic pain’.

Table S3. Factor consumption in participants ≥50 years of age only ever on prophylaxis in A-LONG/ASPIRE and B-LONG/B-YOND

	A-LONG/ASPIRE (n=17)	B-LONG/B-YOND (n=11)
**Factor consumption (IU/kg), mean (SD)**		
At the end of A-LONG/B-LONG (n=17)	52.53 (12.36)	76.52 (28.18)
At the end of ASPIRE/B-YOND (n=17)	58.99 (12.06)	80.40 (29.72)
**Change in factor consumption (IU/kg) from the end of A-LONG/B-LONG to the end of ASPIRE/B-YOND, mean (SD)**	6.46 (10.57)	3.89 (15.65)
**Annualized factor consumption (IU/kg)*, mean (SD)**		
Overall prophylaxis	5001 (1140)	3537 (1060)
Individualized prophylaxis regimen	5391 (1105) [n=13]	3664 (820) [n=7]
Modified prophylaxis regimen	4902 (1741) [n=3]	4013 (1504) [n=3]
Weekly prophylaxis regimen	4234 (406) [n=5]	3890 (1722) [n=6]

*Annualized factor consumption derived as (total dose [IU/kg]) of rFVIIIFc/rFIXFc received during the efficacy period / total number of days in efficacy period)*365.25. Patients may appear in more than 1 treatment regimen; they are considered in each treatment regimen they participated in for the duration of time on that regimen.

Table S4. Safety in participants ≥50 years of age in A-LONG/ASPIRE and B-LONG/B-YOND

	A-LONG/ASPIRE (N=21)	B-LONG/B-YOND (N=26)
**Participants with TEAEs, n (%)**	**20 (95.2)**	**23 (88.5)**
**Number of TEAEs**	**226**	**270**
Mild	158	164
Moderate	47	92
Severe	21	14
**Participants with treatment-related TEAEs, n (%)**	**0**	**3 (11.5)**
**Number of treatment-related TEAEs**	**0**	**3**
**General disorders and administration site conditions**	-	**1**
Non-cardiac chest pain	-	1
**Vascular disorders**	-	**1**
Hypotension	-	1
**Nervous system disorders**	-	**1**
Dysgeusia	-	1
**Participants with serious TEAEs, n (%)**	**8 (38.1)**	**14 (53.8)**
**Number of serious TEAEs**	**25**	**31**
**Participants with treatment-related serious TEAEs**	**0**	**0**
**Deaths, n (%)**	0	0

TEAE, treatment-emergent adverse event.

Table S5. Treatment-emergent adverse events in participants ≥50 years of age during A-LONG/ASPIRE

	Number of patients, n (%)	Number of events
**Infections and infestations**	**16 (76.2)**	**44**
Upper Respiratory Tract Infection	5 (23.8)	10
Influenza	4 (19.0)	6
Hepatitis C	3 (14.3)	4
Nasopharyngitis	2 (9.5)	3
Cellulitis	1 (4.8)	2
Ear Infection	2 (9.5)	2
Pneumonia	1 (4.8)	2
Urinary Tract Infection	2 (9.5)	2
Abscess Jaw	1 (4.8)	1
Acarodermatitis	1 (4.8)	1
Chronic Sinusitis	1 (4.8)	1
Hordeolum	1 (4.8)	1
Infectious Pleural Effusion	1 (4.8)	1
Molluscum Contagiosum	1 (4.8)	1
Otitis Media	1 (4.8)	1
Pericoronitis	1 (4.8)	1
Pharyngitis	1 (4.8)	1
Postoperative Wound Infection	1 (4.8)	1
Rhinitis	1 (4.8)	1
Streptococcal Sepsis	1 (4.8)	1
Wound Infection	1 (4.8)	1
**Injury, poisoning and procedural complications**	**12 (57.1)**	**35**
Fall	8 (38.1)	9
Laceration	3 (14.3)	4
Contusion	3 (14.3)	3
Excoriation	3 (14.3)	3
Limb Injury	3 (14.3)	3
Stress Fracture	1 (4.8)	2
Anemia Postoperative	1 (4.8)	1
Back Injury	1 (4.8)	1
Chest Injury	1 (4.8)	1
Femur Fracture	1 (4.8)	1
Head Injury	1 (4.8)	1
Incision Site Hematoma	1 (4.8)	1
Joint Injury	1 (4.8)	1
Periorbital Hematoma	1 (4.8)	1
Post-Traumatic Pain	1 (4.8)	1
Postoperative Fever	1 (4.8)	1
Procedural Pain	1 (4.8)	1
**Gastrointestinal disorders**	**10 (47.6)**	**30**

	Number of patients, n (%)	Number of events
Vomiting	3 (14.3)	5
Constipation	2 (9.5)	4
Dental Caries	2 (9.5)	4
Diarrhea	4 (19.0)	4
Nausea	2 (9.5)	3
Hemorrhoids	1 (4.8)	2
Abdominal Pain Upper	1 (4.8)	1
Dyspepsia	1 (4.8)	1
Epigastric Discomfort	1 (4.8)	1
Fecaloma	1 (4.8)	1
Food Poisoning	1 (4.8)	1
Gastritis	1 (4.8)	1
Gastritis Hemorrhagic	1 (4.8)	1
Toothache	1 (4.8)	1
**Musculoskeletal and connective tissue disorders**	**10 (47.6)**	**25**
Hemophilic Arthropathy	4 (19.0)	5
Arthralgia	3 (14.3)	4
Osteoarthritis	1 (4.8)	2
Pain In Extremity	2 (9.5)	2
Tenosynovitis	1 (4.8)	2
Back Pain	1 (4.8)	1
Fracture Delayed Union	1 (4.8)	1
Intervertebral Disc Disorder	1 (4.8)	1
Joint Range Of Motion Decreased	1 (4.8)	1
Musculoskeletal Discomfort	1 (4.8)	1
Musculoskeletal Pain	1 (4.8)	1
Musculoskeletal Stiffness	1 (4.8)	1
Myalgia	1 (4.8)	1
Myositis	1 (4.8)	1
Spinal Osteoarthritis	1 (4.8)	1
**General disorders and administration site conditions**	**10 (47.6)**	**14**
Oedema Peripheral	3 (14.3)	3
Pyrexia	3 (14.3)	3
Adverse Drug Reaction	2 (9.5)	2
Influenza Like Illness	2 (9.5)	2
Device Breakage	1 (4.8)	1
Fatigue	1 (4.8)	1
Infusion Site Reaction	1 (4.8)	1
Local Swelling	1 (4.8)	1
**Skin and subcutaneous tissue disorders**	**7 (33.3)**	**14**
Rash	2 (9.5)	3
Pruritus	2 (9.5)	2
Rash Papular	1 (4.8)	2
Urticaria	2 (9.5)	2
Alopecia	1 (4.8)	1

	Number of patients, n (%)	Number of events
Blister	1 (4.8)	1
Dermatitis Contact	1 (4.8)	1
Erythrodermic Psoriasis	1 (4.8)	1
Urticaria Contact	1 (4.8)	1
**Nervous system disorders**	**8 (38.1)**	**13**
Headache	3 (14.3)	4
Restless Legs Syndrome	1 (4.8)	3
Cubital Tunnel Syndrome	1 (4.8)	1
Diabetic Neuropathy	1 (4.8)	1
Dizziness	1 (4.8)	1
Monoplegia	1 (4.8)	1
Nerve Compression	1 (4.8)	1
Sinus Headache	1 (4.8)	1
**Metabolism and nutrition disorders**	**5 (23.8)**	**7**
Diabetes Mellitus	3 (14.3)	3
Dehydration	1 (4.8)	1
Glucose Tolerance Impaired	1 (4.8)	1
Hyperlipidemia	1 (4.8)	1
Hypokalemia	1 (4.8)	1
**Respiratory, thoracic and mediastinal disorders**	**4 (19.0)**	**7**
Nasal Congestion	2 (9.5)	3
Cough	1 (4.8)	1
Dyspnea Exertional	1 (4.8)	1
Oropharyngeal Pain	1 (4.8)	1
Rhinorrhea	1 (4.8)	1
**Investigations**	**4 (19.0)**	**6**
Blood Bilirubin Increased	1 (4.8)	2
Alanine Aminotransferase Increased	1 (4.8)	1
Blood Cholesterol Increased	1 (4.8)	1
Body Temperature Increased	1 (4.8)	1
Platelet Count Increased	1 (4.8)	1
**Renal and urinary disorders**	**6 (28.6)**	**6**
Chromaturia	1 (4.8)	1
Hematuria	1 (4.8)	1
Nephritis	1 (4.8)	1
Nephrolithiasis	1 (4.8)	1
Renal Failure Chronic	1 (4.8)	1
Renal Impairment	1 (4.8)	1
**Neoplasms benign, malignant and unspecified (incl cysts and polyps)**	**5 (23.8)**	**5**
Skin Papilloma	4 (19.0)	4
Skin Neoplasm Bleeding	1 (4.8)	1
**Blood and lymphatic system disorders**	**3 (14.3)**	**4**
Anemia	1 (4.8)	1
Anemia Macrocytic	1 (4.8)	1
Iron Deficiency Anemia	1 (4.8)	1

	Number of patients, n (%)	Number of events
Lymphadenopathy	1 (4.8)	1
**Cardiac disorders**	**3 (14.3)**	**4**
Palpitations	1 (4.8)	2
Atrioventricular Block	1 (4.8)	1
Myocardial Infarction	1 (4.8)	1*
**Ear and labyrinth disorders**	**2 (9.5)**	**3**
Cerumen Impaction	1 (4.8)	1
Ear Congestion	1 (4.8)	1
Inner Ear Inflammation	1 (4.8)	1
**Hepatobiliary disorders**	**2 (9.5)**	**3**
Cholangitis Acute	1 (4.8)	1
Hepatic Cirrhosis	1 (4.8)	1
Jaundice	1 (4.8)	1
**Psychiatric disorders**	**2 (9.5)**	**3**
Insomnia	2 (9.5)	3
**Vascular disorders**	3 (14.3)	**3**
Hypertension	3 (14.3)	3

*The patient had cardiac risk factors and the investigator considered the event “unrelated” to study treatment. The patient continued treatment with rFVIIIFc.

Table S6. Treatment-emergent adverse events in participants ≥50 years of age during B-LONG/B-YOND

	Number of patients, n (%)	Number of events
**Infections and infestations**	**18 (69.2)**	**51**
Nasopharyngitis	7 (26.9)	11
Cellulitis	3 (11.5)	6
Hepatitis C	4 (15.4)	5
Tooth Infection	3 (11.5)	4
Skin Infection	2 (7.7)	3
Bacterial Sepsis	1 (3.8)	2
Ear Infection	1 (3.8)	2
Oral Candidiasis	2 (7.7)	2
Upper Respiratory Tract Infection	2 (7.7)	2
Device Related Infection	1 (3.8)	1
Enterobacter Infection	1 (3.8)	1
Gastroenteritis Viral	1 (3.8)	1
Infected Bites	1 (3.8)	1
Infected Dermal Cyst	1 (3.8)	1
Influenza	1 (3.8)	1
Localized Infection	1 (3.8)	1
Pneumonia	1 (3.8)	1
Sialadenitis	1 (3.8)	1
Staphylococcal Bacteremia	1 (3.8)	1
Streptococcal Infection	1 (3.8)	1
Tooth Abscess	1 (3.8)	1
Urinary Tract Infection	1 (3.8)	1
Wound Infection Staphylococcal	1 (3.8)	1
**Injury, poisoning and procedural complications**	**14 (53.8)**	**48**
Fall	6 (23.1)	17
Ligament Sprain	1 (3.8)	3
Procedural Pain	2 (7.7)	3
Head Injury	2 (7.7)	2
Joint Injury	1 (3.8)	2
Post Procedural Complication	1 (3.8)	2
Rib Fracture	2 (7.7)	2
Anemia Postoperative	1 (3.8)	1
Back Injury	1 (3.8)	1
Ear Canal Injury	1 (3.8)	1
Femoral Neck Fracture	1 (3.8)	1
Joint Dislocation	1 (3.8)	1
Laceration	1 (3.8)	1
Meniscus Lesion	1 (3.8)	1

	Number of patients, n (%)	Number of events
Muscle Injury	1 (3.8)	1
Post Procedural Hematoma	1 (3.8)	1
Post Procedural Edema	1 (3.8)	1
Road Traffic Accident	1 (3.8)	1
Scratch	1 (3.8)	1
Spinal Compression Fracture	1 (3.8)	1
Thermal Burn	1 (3.8)	1
Traumatic Lung Injury	1 (3.8)	1
Traumatic Ulcer	1 (3.8)	1
Wound Complication	1 (3.8)	1
**Gastrointestinal disorders**	**13 (50.0)**	**33**
Nausea	3 (11.5)	5
Diarrhea	2 (7.7)	4
Constipation	3 (11.5)	3
Gastroesophageal Reflux Disease	2 (7.7)	3
Toothache	3 (11.5)	3
Abdominal Discomfort	2 (7.7)	2
Abdominal Pain	2 (7.7)	2
Vomiting	2 (7.7)	2
Dental Caries	1 (3.8)	1
Dysphagia	1 (3.8)	1
Enterocolitis	1 (3.8)	1
Hemorrhoids	1 (3.8)	1
Inguinal Hernia	1 (3.8)	1
Periodontitis	1 (3.8)	1
Small Intestinal Obstruction	1 (3.8)	1
Tooth Disorder	1 (3.8)	1
Upper Gastrointestinal Hemorrhage	1 (3.8)	1
**Musculoskeletal and connective tissue disorders**	**11 (42.3)**	**33**
Arthralgia	4 (15.4)	6
Spinal Column Stenosis	2 (7.7)	6
Hemophilic Arthropathy	3 (11.5)	3
Musculoskeletal Pain	3 (11.5)	3
Neck Pain	2 (7.7)	3
Back Pain	1 (3.8)	2
Pain In Extremity	2 (7.7)	2
Arthropathy	1 (3.8)	1
Hemarthrosis	1 (3.8)	1
Joint Range of Motion Decreased	1 (3.8)	1
Muscle Tightness	1 (3.8)	1
Musculoskeletal Stiffness	1 (3.8)	1
Osteoarthritis	1 (3.8)	1

	Number of patients, n (%)	Number of events
Rotator Cuff Syndrome	1 (3.8)	1
Synovial Cyst	1 (3.8)	1
**Nervous system disorders**	**12 (46.2)**	**27**
Headache	4 (15.4)	4
Tongue Biting	1 (3.8)	4
Phantom Pain	1 (3.8)	3
Dizziness	2 (7.7)	2
Dysgeusia	2 (7.7)	2
Posterior Interosseous Syndrome	1 (3.8)	2
Spinal Cord Paralysis	1 (3.8)	2
Cervicobrachial Syndrome	1 (3.8)	1
Dysarthria	1 (3.8)	1
Hypoesthesia	1 (3.8)	1
Migraine	1 (3.8)	1
Neuralgia	1 (3.8)	1
Neuropathy Peripheral	1 (3.8)	1
Presyncope	1 (3.8)	1
Syncope	1 (3.8)	1
**General disorders and administration site conditions**	**10 (38.5)**	**15**
Edema Peripheral	2 (7.7)	5
Application Site Dermatitis	1 (3.8)	2
Pain	2 (7.7)	2
Chest Discomfort	1 (3.8)	1
Device Breakage	1 (3.8)	1
Influenza Like Illness	1 (3.8)	1
Infusion Site Pain	1 (3.8)	1
Injection Site Inflammation	1 (3.8)	1
Non-Cardiac Chest Pain	1 (3.8)	1
**Skin and subcutaneous tissue disorders**	**8 (30.8)**	**11**
Rash	3 (11.5)	3
Pruritus	2 (7.7)	2
Eczema	1 (3.8)	1
Hyperkeratosis	1 (3.8)	1
Pruritus Allergic	1 (3.8)	1
Rash Maculo-Papular	1 (3.8)	1
Skin Ulcer	1 (3.8)	1
Urticaria	1 (3.8)	1
**Respiratory, thoracic and mediastinal disorders**	**6 (23.1)**	**9**
Cough	2 (7.7)	3
Oropharyngeal Pain	3 (11.5)	3
Asthma	1 (3.8)	1
Dyspnea	1 (3.8)	1

	Number of patients, n (%)	Number of events
Dyspnea Exertional	1 (3.8)	1
**Vascular disorders**	**6 (23.1)**	**9**
Hypertension	4 (15.4)	6
Hypotension	2 (7.7)	2
Orthostatic Hypotension	1 (3.8)	1
**Cardiac disorders**	**3 (11.5)**	**6**
Angina Pectoris	2 (7.7)	3
Coronary Artery Stenosis	1 (3.8)	1
Palpitations	1 (3.8)	1
Tachycardia	1 (3.8)	1
**Psychiatric disorders**	**5 (19.2)**	**6**
Insomnia	3 (11.5)	3
Anxiety	1 (3.8)	1
Sleep Disorder	1 (3.8)	1
Stress	1 (3.8)	1
**Investigations**	**2 (7.7)**	**5**
Heart Rate Increased	1 (3.8)	1
Monocyte Count Increased	1 (3.8)	1
Neutrophil Count Decreased	1 (3.8)	1
Platelet Count Decreased	1 (3.8)	1
White Blood Cell Count Decreased	1 (3.8)	1
**Neoplasms benign, malignant and unspecified (incl cysts and polyps)**	**3 (11.5)**	**4**
Hepatic Neoplasm Malignant	2 (7.7)	3
Skin Papilloma	1 (3.8)	1
**Immune system disorders**	**3 (11.5)**	**3**
Allergy To Arthropod Bite	2 (7.7)	2
Drug Hypersensitivity	1 (3.8)	1
**Reproductive system and breast disorders**	**3 (11.5)**	**3**
Hematospermia	1 (3.8)	1
Prostatism	1 (3.8)	1
Pruritus Genital	1 (3.8)	1
**Blood and lymphatic system disorders**	**2 (7.7)**	**2**
Anemia	2 (7.7)	2
**Metabolism and nutrition disorders**	**1 (3.8)**	**2**
Hyperglycemia	1 (3.8)	1
Hypokalemia	1 (3.8)	1
**Ear and labyrinth disorders**	**1 (3.8)**	**1**
Vertigo	1 (3.8)	1
**Eye disorders**	**1 (3.8)**	**1**
Cataract	1 (3.8)	1
**Renal and urinary disorders**	**1 (3.8)**	**1**
Hematuria	1 (3.8)	1

Table S7. Serious treatment-emergent adverse events in participants ≥50 years of age during A-LONG/ASPIRE

	Number of patients, n (%)	Number of events
**Infections and infestations**	**4 (19.0)**	**7**
Hepatitis C	2 (9.5)	2
Chronic Sinusitis	1 (4.8)	1
Infectious Pleural Effusion	1 (4.8)	1
Pericoronitis	1 (4.8)	1
Pneumonia	1 (4.8)	1
Streptococcal Sepsis	1 (4.8)	1
**Musculoskeletal and connective tissue disorders**	**4 (19.0)**	**7**
Hemophilic Arthropathy	4 (19.0)	5
Intervertebral Disc Disorder	1 (4.8)	1
Spinal Osteoarthritis	1 (4.8)	1
**Gastrointestinal disorders**	**2 (9.5)**	**4**
Constipation	1 (4.8)	2
Gastritis Hemorrhagic	1 (4.8)	1
Vomiting	1 (4.8)	1
**Renal and urinary disorders**	**2 (9.5)**	**2**
Nephrolithiasis	1 (4.8)	1
Renal Impairment	1 (4.8)	1
**Cardiac disorders**	**1 (4.8)**	**1**
Myocardial Infarction	1 (4.8)	1*
**Hepatobiliary Disorders**	**1 (4.8)**	**1**
Cholangitis Acute	1 (4.8)	1
**Injury, Poisoning and Procedural Complications**	**1 (4.8)**	**1**
Femur Fracture	1 (4.8)	1
**Metabolism And Nutrition Disorders**	**1 (4.8)**	**1**
Dehydration	1 (4.8)	1
**Nervous System Disorders**	**1 (4.8)**	**1**
Restless Legs Syndrome	1 (4.8)	1

*The patient had cardiac risk factors and the investigator considered the event “unrelated” to study treatment. The patient continued treatment with rFVIIIFc.

Table S8. Serious treatment-emergent adverse events in participants ≥50 years of age during B-LONG/B-YOND

	Number of patients, n (%)	Number of events
**Infections and infestations**	**5 (19.2)**	**8**
Cellulitis	2 (7.7)	3
Bacterial Sepsis	1 (3.8)	2
Device Related Infection	1 (3.8)	1
Hepatitis C	1 (3.8)	1
Staphylococcal Bacteremia	1 (3.8)	1
**Injury, poisoning and procedural complications**	**4 (15.4)**	**7**
Fall	3 (11.5)	3
Rib Fracture	2 (7.7)	2
Femoral Neck Fracture	1 (3.8)	1
Post Procedural Hematoma	1 (3.8)	1
**Musculoskeletal and connective tissue disorders**	**4 (15.4)**	**5**
Hemophilic Arthropathy	2 (7.7)	2
Arthralgia	1 (3.8)	1
Hemarthrosis	1 (3.8)	1
Spinal Column Stenosis	1 (3.8)	1
**Cardiac disorders**	**2 (7.7)**	**3**
Angina Pectoris	1 (3.8)	2
Tachycardia	1 (3.8)	1
**Gastrointestinal disorders**	**3 (11.5)**	**3**
Enterocolitis	1 (3.8)	1
Inguinal Hernia	1 (3.8)	1
Upper Gastrointestinal Hemorrhage	1 (3.8)	1
**Neoplasms benign, malignant and unspecified (incl cysts and polyps)**	**2 (7.7)**	**3**
Hepatic Neoplasm Malignant	2 (7.7)	3
**General disorders and administration site conditions**	**1 (3.8)**	**1**
Pain	1 (3.8)	1
**Nervous system disorders**	**1 (3.8)**	**1**
Posterior Interosseous Syndrome	1 (3.8)	1
